# Risk of endoscopic biliary interventions in primary sclerosing cholangitis is similar between patients with and without cirrhosis

**DOI:** 10.1371/journal.pone.0202686

**Published:** 2018-08-20

**Authors:** Moritz Peiseler, David Reiners, Hans O. Pinnschmidt, Marcial Sebode, Franziska Jung, Johannes Hartl, Roman Zenouzi, Hanno Ehlken, Stefan Groth, Guido Schachschal, Thomas Rösch, Christina Weiler-Normann, Ansgar W. Lohse, Christoph Schramm

**Affiliations:** 1 I. Department of Medicine, University Medical Centre Hamburg-Eppendorf, Hamburg, Germany; 2 Department of Medical Biometry and Epidemiology, University Medical Centre Hamburg-Eppendorf, Hamburg, Germany; 3 Department of Interdisciplinary Endoscopy, University Medical Centre Hamburg-Eppendorf, Hamburg, Germany; 4 Martin Zeitz Centre for Rare Diseases, University Medical Centre Hamburg-Eppendorf, Hamburg, Germany; Texas A&M University, UNITED STATES

## Abstract

**Background:**

Endoscopic retrograde cholangiography (ERC) is a mainstay of therapy in patients with primary sclerosing cholangitis (PSC) and obstructive cholestasis. Patients with liver cirrhosis have an increased risk of surgical complications and are more susceptible to infections. Since PSC often progresses to cirrhosis, we aimed to assess whether ERC is associated with increased risk of complications in patients with PSC and cirrhosis.

**Methods:**

Out of 383 patients with PSC, 208 patients received endoscopic treatment between 2009–2017. Seventy patients had cirrhosis when ERC was performed and 138 patients had no signs of cirrhosis. Overall, 663 ERC procedures were analysed, with 250 ERC in patients with cirrhosis and 413 ERC in patients without cirrhosis. Data were analysed retrospectively from a prospectively acquired database using repeated measures logistic regression.

**Results:**

Overall, 40 procedure-related complications were documented in 663 ERC interventions (6%). The rate of complications was similar between patients with and without cirrhosis (4.4% vs. 7.0%). First-time ERC was associated with a higher risk of complications (17.5% vs. 4.9%). Biliary sphincterotomy, stent placement and female sex, but not presence of liver cirrhosis, were identified as risk factors for overall complications in multivariate analysis. Patients without cirrhosis showed a significant decline of ALP and bilirubin levels after the first two interventions. In contrast, in patients with cirrhosis, ALP and bilirubin levels did not significantly decline after ERC.

**Conclusions:**

In patients with PSC, cirrhosis was not a risk factor for post-ERC complications. Therefore, cirrhosis should not preclude endoscopic intervention in patients with clear clinical indication.

## Introduction

Primary sclerosing cholangitis (PSC) is a chronic inflammatory disease of intra- and extrahepatic bile ducts with characteristic strictures and bile duct obstruction [1]. PSC progresses to end-stage liver disease necessitating organ transplantation. Clinical symptoms include right upper quadrant abdominal pain, pruritus, jaundice, cholangitis, fatigue, but at least 50% of PSC patients are asymptomatic at initial presentation [2, 3]. Magnetic resonance imaging has replaced endoscopic retrograde cholangiography (ERC) as diagnostic modality of choice [4]. ERC with biliary interventions is currently recommended in symptomatic patients, in case of rapid increase in cholestatic liver enzymes and in patients with progressive dominant strictures on imaging [5–8]. Treatment of dominant strictures with balloon or bougie dilation is frequently performed to relieve symptoms and improve biliary drainage. Although clinical practice in most centres, to date there are no controlled studies demonstrating the efficacy of stricture dilation on disease progression [8].

There is paucity of data regarding biliary interventions in patients with PSC and established cirrhosis and it is unclear, whether benefit outweighs risk [8]. Patients with liver cirrhosis have an increased risk following surgical procedures and anesthesia and also suffer from high susceptibility of infections [9–11]. Therefore, patients with PSC and established cirrhosis might also have an increased risk for adverse events associated with endoscopic therapy and there is lack of data on the risk of this subgroup. A study performed in North America between 1980 and 1994 reported on 10 patients with PSC and cirrhosis with endoscopic or percutaneous stricture treatment and found no change in baseline bilirubin before and one year after intervention [12]. Another study from Germany reported on deterioration of laboratory values of cholestasis following diagnostic ERC in seven of eight patients with stage III or IV fibrosis compared to patients with early stages of fibrosis [13].

Since to date differences in adverse events between cirrhotic and non-cirrhotic PSC patients have not been analysed, the aim of this study was to assess the safety of ERC in a large single-centre cohort of PSC patients with and without established cirrhosis. Our findings indicate that in patients with PSC and established cirrhosis ERC could be safely performed and was not associated with increased risk of adverse events.

## Patients and methods

This is a single-centre analysis of a tertiary centre, including 383 patients with PSC managed at the I. Department of Medicine, University Medical Centre Hamburg-Eppendorf from 2009–2017. Data were collected prospectively in a database and analysed retrospectively. Written informed consent was obtained from each patient and the local ethics committee approved of the study (PV4081-003; Ethics Committee of the Medical Association Hamburg, Germany). The study conforms with the ethical guidelines of the Declaration of Helsinki and its revised versions. Diagnosis of PSC was made using a combination of clinical, biochemical and cholangiographic (magnetic resonance cholangiopancreatography and / or endoscopic retrograde cholangiopancreatography) features according to recent guidelines [5–8]. Diagnosis of cirrhosis was based on the presence of at least one of the following characteristics: ascites, gastroesophageal varices or hepatic encephalopathy, liver histology demonstrating cirrhosis or transient elastography ≥ 14.4 kPa, as previously validated as an appropriate cut-off value for cirrhosis [14]. Patients with secondary sclerosing cholangitis or IgG4-associated cholangitis were excluded. Follow up information was obtained by reviewing each patient’s chart. Patients were routinely admitted to the ward to be monitored for 24–48 hours following endoscopic intervention. All patients were scheduled for a routine consultation at the outpatient hepatology service (YAEL-Centre for Autoimmune Liver Diseases) 4–12 weeks after endoscopy to discuss treatment efficacy and adverse events.

Procedure-associated adverse events were classified in accordance with the most recent guideline of the American Society for Gastrointestinal Endoscopy (ASGE) [15]. Post-ERC pancreatitis was defined by new onset of abdominal pain and a ≥ 3-fold elevation of serum lipase levels for up to two weeks after the procedure. Post-ERC cholangitis was defined by fever, leukocytosis and / or positive blood culture necessitating the use of antibiotics. Bleeding was defined if haemorrhage was leading to blood transfusion or re-intervention. Perforation was defined as extravasation of contrast material on radioscopy requiring re-intervention and / or stenting.

Laboratory parameters including alkaline phosphatase (ALP), serum bilirubin and aminotransferase levels as surrogates of cholestasis were assessed before and up to three months after each intervention. If indication for biliary intervention was clinical symptoms of cholestasis (pruritus, right upper quadrant abdominal pain, jaundice), symptoms were graded as improved, stable or worsened retrospectively during follow up visit within three months.

Most patients (96%) received peri-interventional antibiotics, mainly ampicillin/sulbactam i.v., during each procedure after sampling of bile fluid as recommended (6, 8) and was continued orally for two days. Guide wire cannulation without prior contrast was the method of choice for accessing the common bile duct. ERC was routinely performed using propofol sedation in prone position by an experienced endoscopist.

Continuous data are expressed as the median or mean with range. Laboratory values are expressed as mean ± standard deviation. Statistical comparison of serum ALP, bilirubin and aminotransferase levels before and after endoscopic biliary intervention was performed using Wilcoxon signed-rank test after normality distribution was excluded. Because many patients had repeated interventions, repeated measures logistic regression was employed to examine risk factors for the adverse events “overall complications”, “post-ERC pancreatitis” and “post-ERC cholangitis” in uni- and multivariate analyses. The independent variables "sex”, “cirrhosis”, “biliary sphincterotomy”, “stent placement”, “first ERC”, “UDCA therapy”, “immunosuppressive therapy”, “inflammatory bowel disease” and “cholangiocarcinoma” were considered fixed effects while data on outcome variables gathered at individual interventions per patient were considered repeated measures. Interactions between the independent variable “cirrhosis” and all other independent variables were tested and none was found significant. For multivariate analyses, all independent variables were forced into the regression model. The resulting odds ratios with 95% confidence intervals and p-values are presented. Marginal frequencies estimated from the multivariate analyses are also presented. P-values < 0.05 were considered significant. The statistical packages SPSS® version 25 (IBM Corp., New York, USA) and GraphPad Prism 5.0 (GraphPad Software, Inc., San Diego, USA) were used for statistical analyses.

## Results

### Patient characteristics

**Fig 1:**
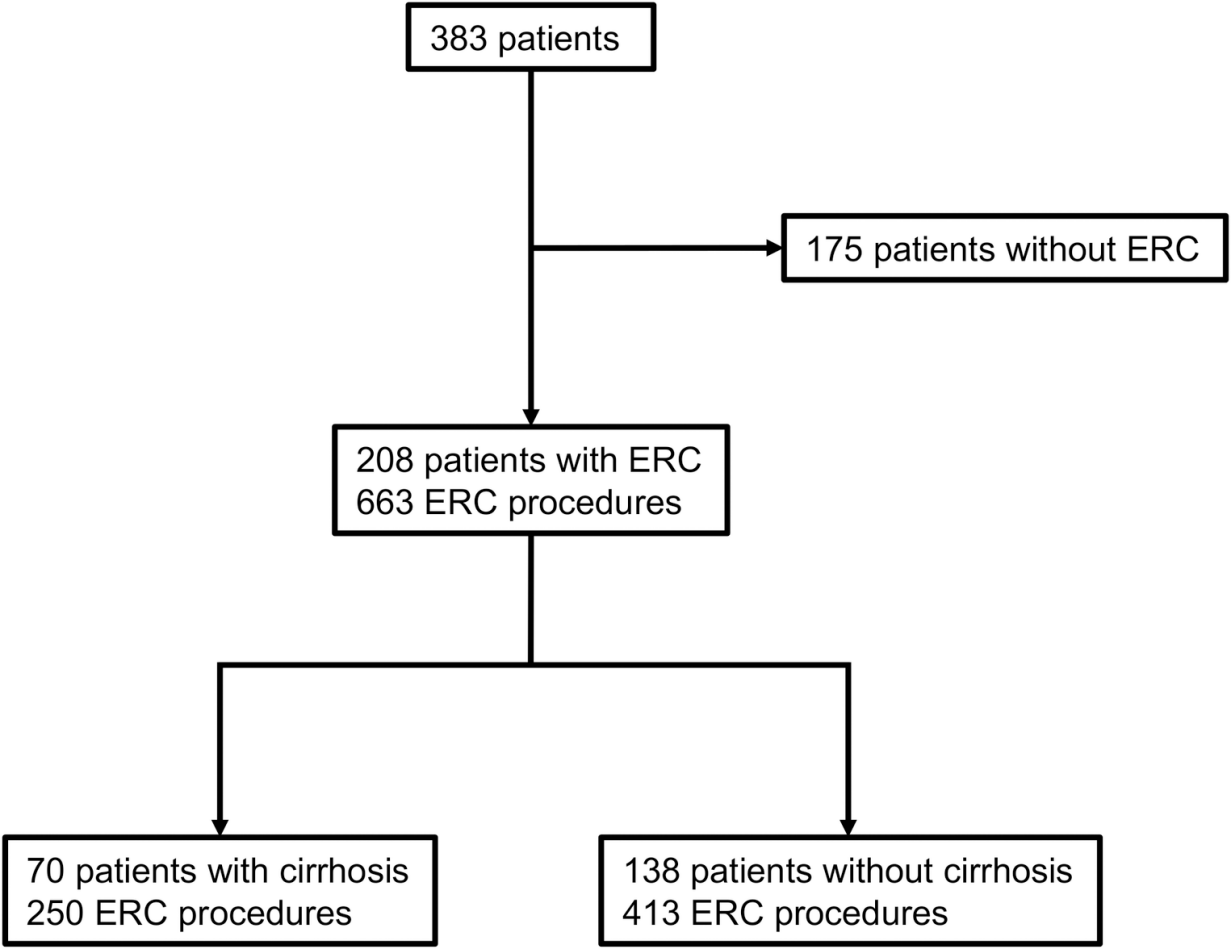
Flow chart of all patients included in the analysis. Out of a total of 383 patients with PSC managed at our centre, 208 patients received at least one ERC between 2009–2017. In 70 patients with 250 procedures, cirrhosis was detected. Another 138 patients with 413 ERC procedures had not progressed to cirrhosis.

Of the whole cohort of 383 patients, 208 (54%) patients received at least one ERC between the years 2009–2017. We evaluated a total of 663 ERC with 413 procedures in 138 patients without cirrhosis and 250 procedures in 70 patients with established cirrhosis (Fig 1). The group of patients with cirrhosis consisted of 34 patients with Child-Pugh class A, 32 with Child-Pugh class B and 4 patients with Child-Pugh class C. In 27 patients, cirrhosis was confirmed histologically, in 34 patients TE was > 14.4 kPa and a total of 39 patients had clinical signs at diagnosis of cirrhosis. Clinical characteristics can be found in Table 1 and S1 Table. Two patients were lost to follow-up. Treatment was unchanged between the time of ERC and follow-up outpatient assessment.

**Table 1:** Patient demographics.

	**PSC with cirrhosis**	**PSC without cirrhosis**	**P-value**
Total number of patients	70	138	
Total number of ERC (N)	250	413	
No. ERC per patient	3.5 (1–14)	3.0 (1–16)	0.058
Male / female	45 (64%) / 25 (36%)	89 (64%) / 49 (36%)	0.999
Mean age at last follow up (yrs.)	46.1 (25–76)	44.5 (30–79)	0.399
Mean age at diagnosis (yrs.)	33.2 (12–68)	36.4 (6–63)	0.051
Observation period after ERC (mos.)	28 (2–88)	33 (2–91)	0.249
Time to last follow up (yrs.)	12.5 (0.5–36)	8 (0.1–35)	< 0.001
PSC-associated IBD	41 (60%)	87 (63%)	0.549
UDCA treatment	61 (87%)	119 (86%)	0.999
Immunosuppression	28 (40%)	66 (48%)	0.305

ERC = endoscopic retrograde cholangiography. IBD = inflammatory bowel disease. UDCA = ursodesoxycholic acid.

### Indications for biliary intervention

In the group of patients with cirrhosis, ERC was performed in 38% (96) of interventions due to progressive strictures on imaging, in 31% (78) because patients complained of clinical symptoms and in 21% (53) of cases because of rising cholestatic liver enzymes. Other indications were extraction of previously placed stents, bile duct stone removal and 4% were intended for diagnostic purpose only. In the group of patients without cirrhosis, in 55% (227) of interventions, ERC was performed due to progressive strictures on imaging, in 12% (50) of cases because patients complained of clinical symptoms and in 16% (65) of cases because of rising cholestatic liver enzymes. Other indications were stent extraction, bile duct stone removal and 3% were diagnostic. In the group of patients with cirrhosis, ERC was performed more frequently due to clinical symptoms (31% vs. 12%) and less often because of progressive strictures (38% vs. 55%), indicating reluctance to perform ERC in cirrhotic patients on the basis of imaging or biochemistry alone.

### Details on ERC procedures

In the patients with cirrhosis, biliary sphincterotomy was performed in 14% of interventions (36 / 250) and in 71% (5 / 7) during first-time ERC (Table 2). In the group of patients without cirrhosis, biliary sphincterotomy was performed in 80 interventions (19%). In this group, 50 first-time ERC were performed and biliary sphincterotomy was performed in 78% (39 / 50) of first-time procedures (Table 2). Rates of balloon dilatation, bougie dilatation, stent placement were similar between the cirrhotic and non-cirrhotic groups (Table 2). There were significantly more brush cytologies and forceps biopsies in the group of patients without cirrhosis (Table 2).

**Table 2:** Details on ERC interventions.

	**All patients**	**PSC with cirrhosis**	**PSC without cirrhosis**	**P-value**
Total number of ERC (N)	663	250	413	
Diagnostic ERC	24 (3.6%)	10 (4%)	14 (3%)	0.674
Biliary sphincterotomy	116 (17%)	36 (14%)	80 (19%)	0.114
Balloon dilation	446 (67%)	168 (67%)	277 (67.1%)	0.999
Bougie dilation	124 (18.7%)	40 (16%)	84 (20.3%)	0.182
Stent placement	69 (10.4%)	23 (9.2%)	46 (11.1%)	0.512
Brush cytology	167 (25%)	47 (19%)	120 (29%)	0.003
Forceps biopsy	39 (5.8%)	8 (3%)	31 (7%)	0.026
Periinterventional antibiotics	636 (96%)	240 (96%)	396 (95.9%)	0.999

ERC = endoscopic retrograde cholangiography.

### Adverse events in 663 ERC procedures

Analysing 663 procedures, we documented 40 procedure-associated adverse events giving an overall complication rate of 6% in our cohort. The overall rate of adverse events was not elevated in the group of patients with cirrhosis compared to the group of patients without cirrhosis (4.4% vs. 7.0%) (Table 3). Post-ERC pancreatitis occurred after 12 (2.9%) ERC without cirrhosis and after three (1.2%) ERC with cirrhosis. Post-ERC cholangitis was noted after ten (2.4%) interventions in patients without cirrhosis and after five (2%) interventions in patients with cirrhosis. Bile duct perforation occurred during six (1.4%) procedures in the non-cirrhotic and two procedures (0.8%) in the cirrhotic group. There was one bleeding recorded in the patients without cirrhosis (0.2%) and one bleeding (0.4%) in the patients with cirrhosis (Table 3).

**Table 3:** Adverse events in patients with and without cirrhosis.

	All patients	PSC with cirrhosis	PSC without cirrhosis
Number of patients	208	70	138
Total number of ERC (N)	663	250	413
First-time ERC	57 (8.6%)	7 (2.8%)	50 (12.1%)
Following ERC	606 (91.4%)	243 (97.2%)	363 (87.9%)
Overall adverse events	40 (6.0%)	11 (4.4%)	29 (7.0%)
Post-ERC pancreatitis	15 (2.2%)	3 (1.2%)	12 (2.9%)
Post-ERC cholangitis	15 (2.2%)	5 (2.0%)	10 (2.4%)
Perforation	8 (1.2%)	2 (0.8%)	6 (1.5%)
Bleeding	2 (0.3%)	1 (0.4%)	1 (0.2%)
Adverse events first-time ERC	10 (17.5%)	2 (28.5%)	8 (16.0%)
Post-ERC pancreatitis	8 (14.0%)	1 (14.2%)	7 (14.0%)
Post-ERC cholangitis	0 (0%)	0 (0%)	0 (0%)
Perforation	2 (3.5%)	1 (14.2%)	1 (2.0%)
Bleeding	0 (0%)	0 (0%)	0 (0%)
Adverse events following ERC	30 (4.9%)	9 (3.7%)	21 (5.8%)
Post-ERC pancreatitis	7 (1.1%)	2 (0.8%)	5 (1.4%)
Post-ERC cholangitis	15 (2.5%)	5 (2.0%)	10 (2.8%)
Perforation	6 (0.9%)	1 (0.4%)	5 (1.4%)
Bleeding	2 (0.3%)	1 (0.4%)	1 (0.3%)

ERC = endoscopic retrograde cholangiography.

### Risk factors for ERC in the whole cohort of PSC patients

Since the group of patients with or without cirrhosis may be differently affected by confounders, we next aimed to analyse risk factors associated with procedure related complications. Univariate analysis indicated placement of a temporary stent, biliary sphincterotomy, first-time ERC and female sex as significant risk factors for “overall complications” while for “post-ERC pancreatitis”, only biliary sphincterotomy, first-time ERC and female sex were significant risk factors (Table 4). Significant risk factors for “post-ERC cholangitis” were stent placement and female sex. The complications “bleeding” and “perforation” did not show any significant associations with independent variables, likely due to the low number of events.

**Table 4:** Univariate and multivariate analysis of risk factors for post-ERC adverse events.

Dependent variable	Covariable	Univariate analysis			Multivariate analysis		
		OR	95% CI	p	OR	95% CI	p
	Cirrhosis	0.61	0.27–1.36	0.226	0.79	0.34–1.83	0.589
	First ERC	4.08	1.89–8.85	<0.001	1.59	0.61–4.14	0.342
**Adverse event**	Sphincterotomy	4.38	2.25–8.53	<0.001	3.82	1.82–8.03	<0.001
	Female	2.35	1.14–4.83	0.022	2.26	1.09–4.69	0.030
	Stent	2.49	1.13–5.49	0.024	2.48	1.09–5.65	0.030
	Cirrhosis	0.41	0.11–1.47	0.170	0.88	0.23–3.45	0.857
	First ERC	14.0	5.20–37.51	<0.001	3.18	0.92–10.95	0.067
**Pancreatitis**	Sphincterotomy	20.9	6.24–70.1	<0.001	13.3	3.43–51.9	<0.001
	Female	3.39	1.09–10.5	0.036	3.57	1.20–10.6	0.022
	Stent	2.03	0.53–7.66	0.295	2.00	0.50–8.07	0.329
	Cirrhosis	0.82	0.27–2.51	0.731	0.83	0.27–2.59	0.748
	First ERC	n/a	n/a	n/a	n/a	n/a	n/a
**Cholangitis**	Sphincterotomy	0.72	0.15–3.40	0.679	1.31	0.27–6.42	0.739
	Female	3.39	1.07–10.7	0.040	3.18	1.01–10.01	0.051
	Stent	4.19	1.27–13.8	0.019	3.81	1.07–13.49	0.038
	Cirrhosis	0.55	0.11–2.67	0.456
	First ERC	3.64	0.72–18.3	0.118
**Perforation**	Sphincterotomy	2.88	0.68–12.13	0.150
	Female	0.98	0.23–4.84	0.977
	Stent	n/a	n/a	n/a
	Cirrhosis	1.65	0.10–26.51	0.722
	First ERC	n/a	n/a	n/a
**Bleeding**	Sphincterotomy	4.74	0.30–75.2	0.269
	Female	n/a	n/a	n/a
	Stent	8.05	0.49–131.0	0.143

In multivariate analyses, the independent variables biliary sphincterotomy, stent placement and female sex were indicated as significant risk factors for “overall complications” while for “post-ERC pancreatitis”, significant risk factors were biliary sphincterotomy and female sex (Table 4). For “post-ERC cholangitis” temporary stent placement was a significant risk factor. Of note, liver cirrhosis did not show any significant correlation with post-ERC complications observed.

Additionally, we tested presence of cholangiocarcinoma, treatment with UDCA, immunosuppressive treatment and presence of IBD as potential risk factors but none of these were found significantly associated with adverse events on univariate and multivariate analyses (S2 Table).

### Estimated marginal frequencies

To adjust for potential confounders which could affect the risk of complications in the two groups of patients, we performed an estimation of marginal frequencies. The estimated marginal frequencies of overall complications were 10.7 for patients with cirrhosis and 13.1% for patients without cirrhosis (S3 Table). In case of sphincterotomy, it was 20.9% (vs. 6.5% without) and for female patients 16.8% and 8.3% for male patients respectively. For patients with and without stent placement, it was 17.5% and 7.9%. The estimated marginal frequencies of overall complications were thus significantly elevated for the risk factors female sex, sphincterotomy and stent placement, compared to absence of these risk factors (S2 Table; see Table 4 for p-values).

### Effect of ERC in cirrhotic and non-cirrhotic PSC patients on liver biochemistry

**Fig 2:**
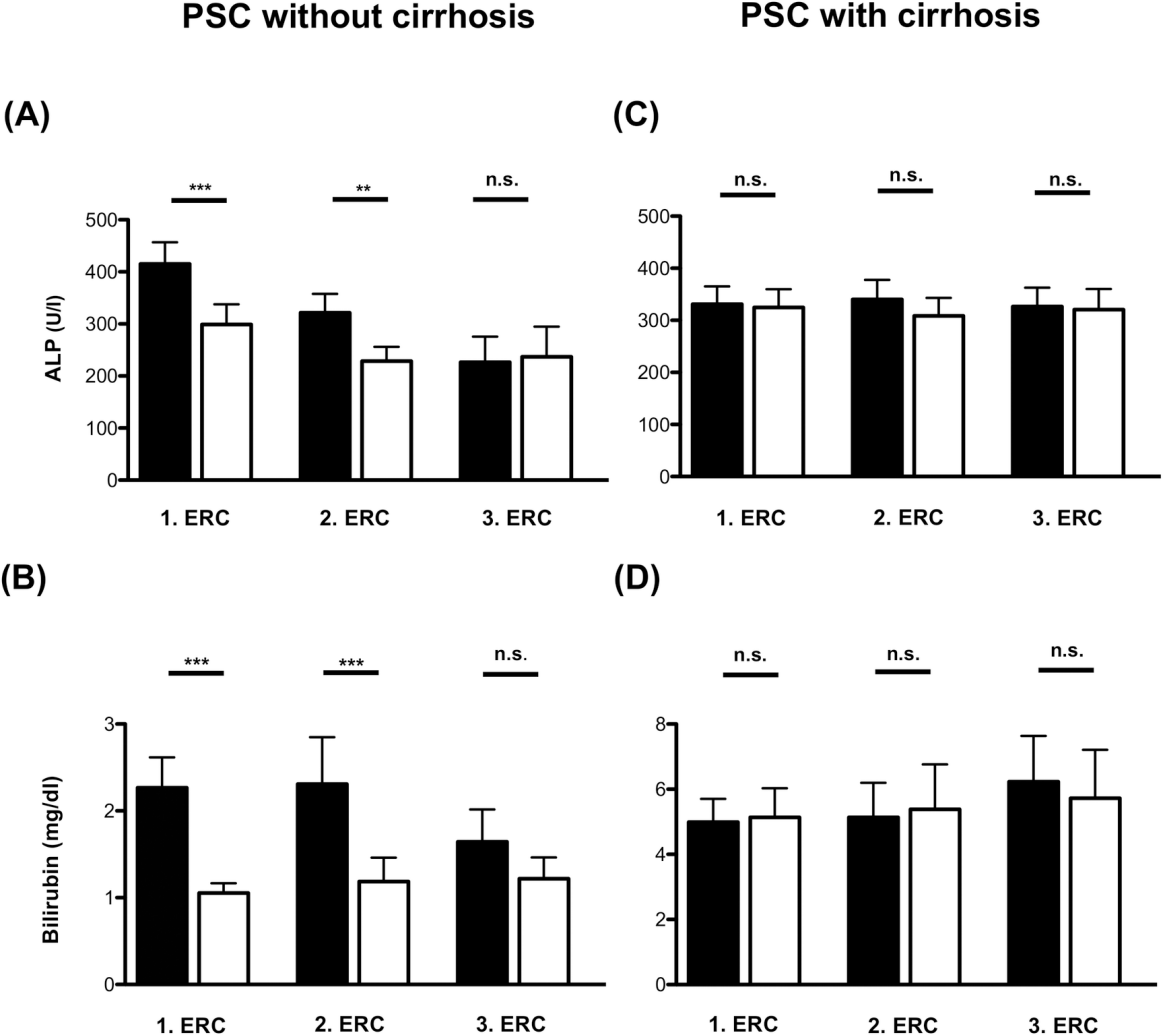
Serum ALP and bilirubin levels before and after endoscopic intervention. Mean alkaline phosphatase (ALP) and bilirubin serum levels of patients without cirrhosis **(A, B)** and with cirrhosis **(C, D)** before and up to three months after therapeutic ERC. ERC = endoscopic retrograde cholangiography. *** P < 0.0001; ** p < 0.001; n.s. = not significant.

In the group of patients without cirrhosis, ALP levels significantly declined after the first (415 U/l ± 271 to 299 U/l ± 249; p = 0.0003) and second intervention (321 U/l ± 201 to 228 U/l ± 153; p = 0.001) (Fig 2A). A third intervention and all following interventions did not significantly improve ALP levels; however, a trend to ALP reduction could still be observed (Fig 2A). Bilirubin levels equally declined in patients without cirrhosis after the first (2.3 mg/dl ± 2.4 to 1.0 mg/dl ± 0.8; p = 0.0003) and second intervention (2.3 mg/dl ± 3.3 to 1.1 mg/dl ± 1.7; p = 0.0007). Thereafter, bilirubin levels were not significantly altered following ERC, similar to ALP levels (Fig 2B). Similarly, levels of AST and ALT were significantly reduced following a first and second intervention in the patients without cirrhosis (S1 Fig).

In contrast, in 70 patients with established cirrhosis, ALP levels did not significantly change after the first (331 U/l ± 167 to 334 U/l ± 202 p = 0.88) or second intervention (340 U/l ± 179 to 309 U/l ± 166; p = 0.45) and after all following interventions (Fig 2C). The same observation was made for serum bilirubin levels after the first intervention (4.9 mg/dl ± 4.8 to 5.1 mg/dl ± 6.1; p = 0.70) and all following interventions (Fig 2D). Analyzing AST and ALT levels in the patients with cirrhosis, we found no change in AST in the patients without cirrhosis throughout the first three interventions and only a significant improvement of ALT after the first ERC and no change in following interventions (S1 Fig).

### Effect of ERC in cirrhotic and non-cirrhotic PSC patients on symptoms

In 128 out of 663 interventions (19%), ERC was performed due to clinical symptoms. In the group of patients with cirrhosis, symptom relief was achieved by 49 interventions (63%), symptoms remained stable in 26 cases (33%) with two patients (2%) describing worsened complaints after ERC. In one patient information was missing. In the group without cirrhosis, symptom relief was achieved by 34 interventions (68%), symptoms remained stable in ten (20%) and worsened in six cases (12%). Therefore, the rate of symptomatic relief was similar between patients with PSC with or without cirrhosis.

## Discussion

Primary sclerosing cholangitis is a challenging disease due to lack of medical therapy and frequent progression to liver cirrhosis. Biliary intervention with mechanical dilation of obstructed bile ducts remains a mainstay of therapy in patients with dominant stenoses and especially in patients presenting with symptoms of obstructive cholestasis. Considering the increased risk of patients with cirrhosis following surgical and anaesthetic procedures [16] and an increased risk of infections in patients with cirrhosis [11], many experts are reluctant to perform ERC in patients with established cirrhosis [8]. However, there is a lack of data on the risk of ERC in PSC patients with established liver cirrhosis. In this large single-centre analysis, we demonstrated that ERC with biliary intervention was safe in patients who have progressed to liver cirrhosis: the rate of adverse events was similar between PSC patients with and without cirrhosis. This is in line with a recent multicentre retrospective study from North America demonstrating a lower rate of adverse events in patients with PSC and cirrhosis compared to cirrhosis of other aetiology following ERC [17]. Furthermore, with an observed rate of adverse events of 6%, ERC was a safe procedure in our cohort, a rate comparable to published data from high volume centres [18–20]. Several studies have evaluated adverse events following ERC in patients with PSC [19–28] reporting heterogeneous results. Overall, patients with PSC seem to have an increased rate of complications [8]. However, not all studies could confirm this finding [25, 28]. A large European study on safety of ERC in patients with PSC showed an overall rate of adverse events of 9% [19] and recently, a retrospective study from North America demonstrated an overall complication rate of 4.3% [20]. In summary, the risk of adverse events in patients with PSC ranges from 1.8–18.4% [8], which is higher compared to ERC for other indications [18].

In our cohort, patients with first-time ERC had a higher rate of adverse events. The rate of biliary sphincterotomy was high during first-time ERC, reflecting difficulty to cannulate the common bile duct in PSC and the intention to lower the risk for subsequent interventions [17, 19, 20]. The low rate of patients with adverse events after following interventions indicates that biliary sphincterotomy indeed could be protective for future interventions. Of note, first-time ERC was a significant risk factor on univariate, but not multivariate analysis. This discrepancy is caused by confounding of first-time ERC with other independent variables, e. g. sphincterotomy. Multivariate analysis adjusts for this confounding, thus yielding more credible results than univariate analysis regarding the actual “net” effect of individual independent variables. Risk factors significantly associated with adverse events identified in our study include female sex, biliary sphincterotomy and placement of a temporary stent. These risk factors have previously been identified and do not seem to be disease specific for PSC [8].

There is debate whether balloon dilation alone or temporary stenting should be the method of choice for treating strictures. Results of a recently published randomized controlled clinical trial comparing balloon dilatation with short-term stenting found increased complication rates after stenting [29]. In our study, we could confirm that interventions with stent placement carry a higher risk for adverse events on univariate and multivariate analysis. However, since in our centre, stent placement is not performed routinely and is only chosen if a complicated stenosis necessitates stenting, selection bias was probably introduced. The overall rate of post-ERC cholangitis was low in our cohort. We routinely administer i.v. antibiotics during the ERC procedure after the bile duct has been cannulated and bile fluid obtained for microbiological culture and on two days orally thereafter. This is of note since it offers the opportunity to assess the bacterial / fungal spectrum in bile which can then be used for targeted treatment in case of cholangitis. Clinical relevance of isolating biliary pathogens during ERC has been previously demonstrated with regards to the choice of subsequent antimicrobial treatment [30].

There are no controlled trials assessing potential efficacy of endoscopic treatment in patients with PSC and no long-term observational studies suggesting a benefit regarding progression to cirrhosis or its complications [8]. In our study, ERC with biliary intervention led to a significant short-term improvement of ALP, bilirubin and aminotransferase levels in patients without cirrhosis after the first and second ERC. In contrast, in cirrhotic patients we did not observe a significant reduction of cholestatic liver enzymes. However, if this short-term improvement of surrogate markers translates into a positive effect on the long-term prognosis can only be answered with prospective studies head-to-head comparing endoscopic therapy with conservative management and should not be concluded from our study. In patients complaining of symptoms such as pruritus, cholangitis or abdominal pain, ERC is performed to achieve symptom relief. In this subgroup, we observed improvement of symptoms in 64–68% of cases, with relief of symptoms in a similar proportion of patients with and without cirrhosis.

Due to the retrospective study design, we did not formally assess symptoms prior or after endoscopy in a standardized fashion introducing a reporting bias. However, since all patients were followed up routinely in our outpatient hepatology service we think that it is unlikely that major adverse events were missed. Furthermore, we used development of laboratory values as a surrogate to assess efficacy of endoscopic treatment. Many other groups reporting on therapeutic ERC have previously relied on biochemical markers and the Mayo Score has been used to assess the efficacy of endoscopic treatment [31, 32]. We did not use the Mayo Score to calculate improved patient outcome after endoscopic intervention, since this score has been developed to assess the natural history of PSC and contains serum bilirubin values, which as we confirm, will be lowered by dilation therapy. In addition, fluctuating cholestatic liver enzymes have been reported even without endoscopic intervention [33]. Furthermore, patients received multiple ERC and it is possible, that patients with favourable outcome were selected for repeated ERC. We employed sophisticated statistical analysis using estimated marginal frequencies to account for these confounders. Our results suggest, that we and others might have underestimated the true rate of adverse events if all confounders were evenly distributed. However, our results consistently show that patients with cirrhosis did not have an increased risk of complications.

To the best of our knowledge, this is the first study comparing safety and potential markers of efficacy in PSC patients with and without established cirrhosis. The results suggest that in non-cirrhotic patients with PSC, endoscopic intervention can lead to improvement in symptoms and surrogate markers of PSC activity and prognosis. In PSC patients with established cirrhosis, endoscopic intervention seems to be safe, but may not impact on prognostic markers. We suggest that endoscopic intervention in PSC cirrhosis should be restricted to patients with symptoms, obstructive stenoses and suspicion of bacterial / fungal cholangitis or for tissue sampling in patients with suspicion of cholangiocarcinoma. Our findings come from a highly specialized group and expert centre and must therefore not be extrapolated to secondary health care centres or "general gastroenterologists". However, we would make the argument that patients with PSC in an advanced stage should be referred to specialized centres.

## Supporting information

S1 Table Characteristics of PSC patients with cirrhosis at the time of diagnosing cirrhosis. (DOCX)

S2 Table Univariate and multivariate analysis of additional risk factors for post-ERC adverse events. (DOCX)

S3 Table Estimated marginal frequencies for adverse events. (DOCX)

S1 Fig AST and ALT before and after endoscopic therapy. (TIF)

## Abbreviations

ERC: endoscopic retrograde cholangiography
PSC: primary sclerosing cholangitis
ASGE: American Society for Gastrointestinal Endoscopy
ALP: alkaline phosphatase
i.v.: intravenous
UDCA: ursodeoxycholic acid
CI: confidence interval
