# Supplementary material for: Risk of endoscopic biliary interventions in primary sclerosing cholangitis is similar between patients with and without cirrhosis
Source: PLoS One. 2018 Aug 20;13(8):e0202686. doi: 10.1371/journal.pone.0202686 (PMC6101401; doi:10.1371/journal.pone.0202686)
Supplement: S1 Table — (DOCX) [file pone.0202686.s001.docx]

|  | PSC with cirrhosis |
| --- | --- |
| Total number of patients | 70 |
| Histology with definite cirrhosis | 27 / 70 (38.5%) |
| TE > 14 | 34 / 70 (48.6%) |
| Median TE | 27.4 (14,4 – 75) |
| Clinical signs | 39 / 70 (55.7%) |
| Ascites | 10 / 70 (14.2%) |
| Esophageal varices | 20 / 70 (28.6%) |
| Hepatic encephalopathy | 1 / 70 (1.4%) |
| Splenomegaly | 32 / 70 (45.7%) |

**Supplementary Tab. 1**: Characteristics of PSC patients with cirrhosis at the time of diagnosing cirrhosis.
