# Supplementary material for: Risk of endoscopic biliary interventions in primary sclerosing cholangitis is similar between patients with and without cirrhosis
Source: PLoS One. 2018 Aug 20;13(8):e0202686. doi: 10.1371/journal.pone.0202686 (PMC6101401; doi:10.1371/journal.pone.0202686)
Supplement: S2 Table — (DOCX) [file pone.0202686.s002.docx]

**Supplementary Table 2**: Univariate and multivariate analysis of additional risk factors for post-ERC adverse events.

| Dependent variable | Covariable | Univariate analysis | | | Multivariate analysis | | |
| --- | --- | --- | --- | --- | --- | --- | --- |
|  |  | **OR** | **95% CI** | **p** | **OR** | **95% CI** | **p** |
|  | CCA | 1.66 | 0.55 – 4.99 | 0.366 | 0.99 | 0.22 – 4.59 | 0.997 |
|  | IBD | 0.50 | 0.24 – 1.03 | 0.061 | 0.47 | 0.22 – 1.02 | 0.055 |
| Adverse event | UDCA | 0.86 | 0.37 – 2.01 | 0.724 | 1.23 | 0.49 – 3.11 | 0.657 |
|  | IS | 0.82 | 0.41 – 1.67 | 0.590 | 1.18 | 0.52 – 2.71 | 0.687 |
|  | CCA | 1.42 | 0.17 – 11.8 | 0.745 | 1.21 | 0.19 – 7.76 | 0.842 |
|  | IBD | 0.45 | 0.15 – 1.36 | 0.157 | 0.44 | 0.11 – 1.69 | 0.233 |
| Pancreatitis | UDCA | 0.73 | 0.19 – 2.75 | 0.642 | 1.75 | 0.39 – 7.79 | 0.462 |
|  | IS | 0.86 | 0.28 – 2.58 | 0.786 | 1.38 | 0.35 – 5.39 | 0.646 |
|  | CCA | 1.42 | 0.19 – 10.5 | 0.730 | 0.45 | 0.04 – 5.39 | 0.529 |
|  | IBD | 0.45 | 0.15 – 1.35 | 0.155 | 0.52 | 0.16 – 1.68 | 0.276 |
| Cholangitis | UDCA | 2.61 | 0.33 – 20.7 | 0.363 | 2.19 | 0.23 – 20.7 | 0.495 |
|  | IS | 0.65 | 0.21 – 1.99 | 0.454 | 0.71 | 0.23 – 2.23 | 0.557 |
|  | CCA | 2.87 | 0.42 – 19.7 | 0.283 |  |  |  |
|  | IBD | 0.52 | 0.13 – 2.03 | 0.347 |  |  |  |
| Perforation | UDCA | 0.55 | 0.10 – 2.75 | 0.464 |  |  |  |
|  | IS | 0.75 | 0.19 – 2.99 | 0.685 |  |  |  |

CCA = cholangiocarcinoma; IBD = inflammatory bowel disease; UDCA = ursodeoxycholic acid; IS = immunosuppression.
