# Supplementary material for: Risk of endoscopic biliary interventions in primary sclerosing cholangitis is similar between patients with and without cirrhosis
Source: PLoS One. 2018 Aug 20;13(8):e0202686. doi: 10.1371/journal.pone.0202686 (PMC6101401; doi:10.1371/journal.pone.0202686)
Supplement: S3 Table — (DOCX) [file pone.0202686.s003.docx]

**Supplementary Tab. 3**: Estimated marginal frequencies for adverse events.

| Category | Covariate | Adverse event | | | Pancreatitis | | | Cholangitis | | |
| --- | --- | --- | --- | --- | --- | --- | --- | --- | --- | --- |
|  |  | Estimated marginal frequency | 95% CI lower limit | 95% CI upper limit | Estimated marginal frequency | 95% CI lower limit | 95% CI upper limit | Estimated marginal frequency | 95% CI lower limit | 95% CI upper limit |
| Cirrhosis | No | 0.131 | 0.087 | 0.193 | 0.041 | 0.018 | 0.898 | <0.0001 | <0.0001 | <0.0001 |
|  | Yes | 0.107 | 0.047 | 0.227 | 0.036 | 0.008 | 0.142 | <0.0001 | <0.0001 | <0.0001 |
| Sex | Female | 0.168 | 0.106 | 0.256 | 0.070 | 0.030 | 0.154 | <0.0001 | <0.0001 | <0.0001 |
|  | Male | 0.083 | 0.039 | 0.167 | 0.021 | 0.005 | 0.075 | <0.0001 | <0.0001 | <0.0001 |
| Sphicter- | No | 0.065 | 0.032 | 0.128 | 0.011 | 0.003 | 0.045 | <0.0001 | <0.0001 | <0.0001 |
| otomy | Yes | 0.209 | 0.125 | 0.328 | 0.127 | 0.056 | 0.262 | <0.0001 | <0.0001 | <0.0001 |
| Stent | No | 0.079 | 0.047 | 0.128 | 0.027 | 0.012 | 0.061 | <0.0001 | <0.0001 | <0.0001 |
|  | Yes | 0.175 | 0.084 | 0.330 | 0.053 | 0.012 | 0.202 | <0.0001 | <0.0001 | <0.0001 |
| First ERC | No | 0.097 | 0.059 | 0.153 | 0.022 | 0.009 | 0.054 | 0.038 | 0.018 | 0.081 |
|  | Yes | 0.145 | 0.063 | 0.299 | 0.066 | 0.018 | 0.217 | <0.0001 | <0.0001 | <0.0001 |
