# Supplementary figures and images for: Risk of endoscopic biliary interventions in primary sclerosing cholangitis is similar between patients with and without cirrhosis
Source: PLoS One. 2018 Aug 20;13(8):e0202686. doi: 10.1371/journal.pone.0202686 (PMC6101401; doi:10.1371/journal.pone.0202686)

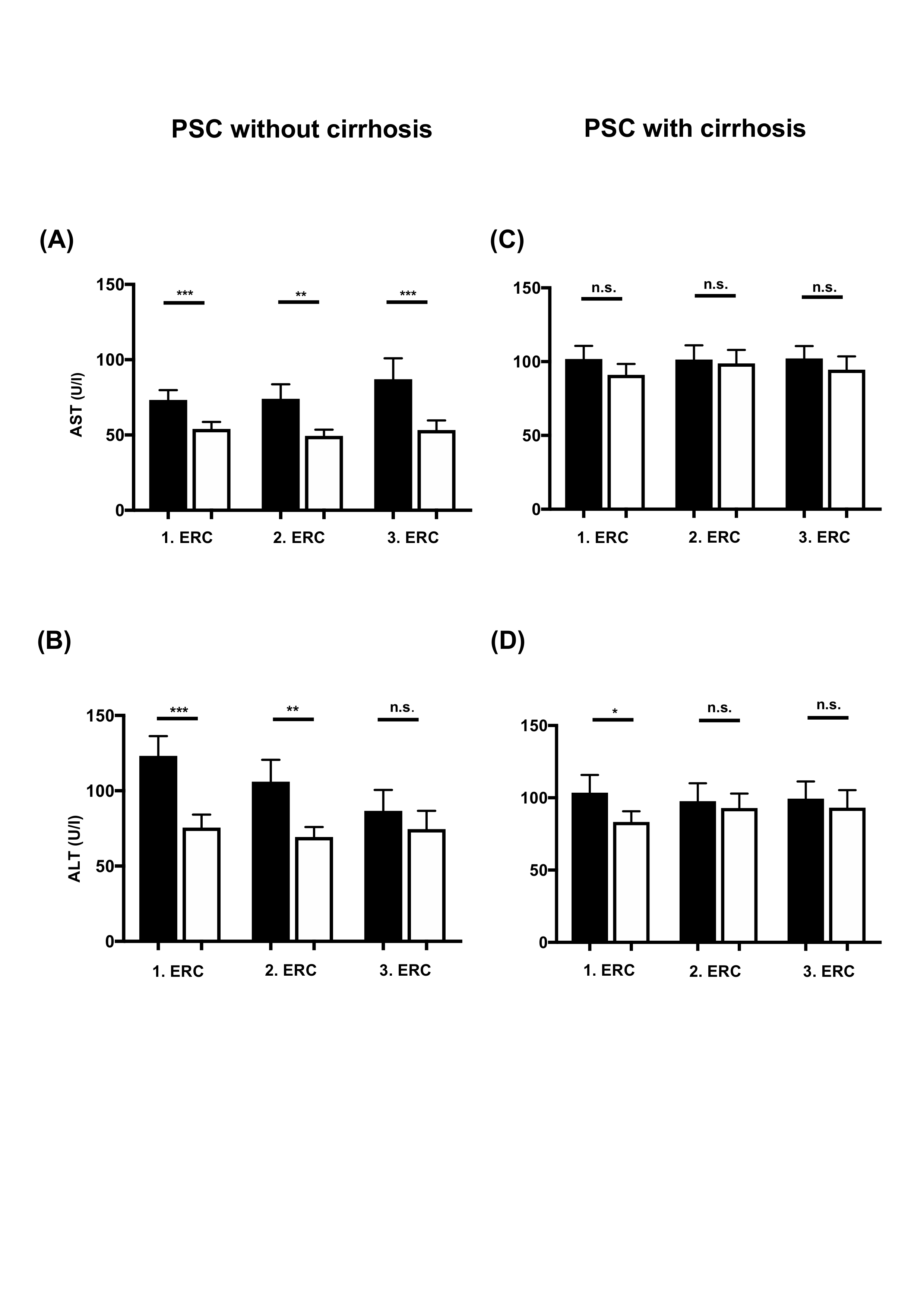

Supplement: S1 Fig — (TIF) [file pone.0202686.s004.tif]
